# An 8-Week Course of *Bifidobacterium longum* 35624^®^ Is Associated with a Reduction in the Symptoms of Irritable Bowel Syndrome

**DOI:** 10.1007/s12602-023-10151-w

**Published:** 2023-09-13

**Authors:** Marion Lenoir, Jörg Wienke, Frédérique Fardao-Beyler, Nadine Roese

**Affiliations:** 1 Biocodex SAS, 7 Avenue Gallieni, F-94257 Gentilly, France; 2 Ritastrasse 2, D-40589 Düsseldorf, Germany; 3 MEDICE Arzneimittel Pütter GmbH & Co. KG, Kuhloweg 37, D-58638 Iserlohn, Germany

**Keywords:** Irritable bowel syndrome, Probiotics, *Bifidobacterium longum* 35624^®^, Post-market clinical follow-up study, Abdominal pain

## Abstract

Irritable bowel syndrome (IBS) is one of the disorders most frequently diagnosed by gastroenterologists. Probiotics are promising tools for the management of IBS. The objective of the present study was to evaluate the effectiveness and tolerability of a probiotic (*Bifidobacterium longum* 35624^®^) in adults (aged 18 or over) with IBS (as defined by the Rome IV criteria). In an open-label, observational, post-market study conducted in Germany, adults with IBS and a prior recommendation for the intake of *B. longum* 35624^®^ were recruited by family physicians. During the 8-week course of treatment, the study participants filled out a weekly questionnaire that enabled calculation of a total IBS symptom score (TISS, the sum of abdominal pain, bloating, passage of gas, constipation, and diarrhea individual symptom scores) and the well-known IBS severity scoring system (IBS-SSS) score. Thirty-seven patients were included. The course of *B. longum* 35624^®^ was associated with a significant reduction (43.4%) in the TISS vs. baseline. The mean individual symptom grades for passage of gas and bloating fell significantly from “moderate” at baseline to “very mild to mild” after 8 weeks of treatment, whereas those for abdominal pain and diarrhea fell significantly from “mild to moderate” to “very mild to mild.” Over 60% of the participants achieved clinically meaningful reductions in the TISS (> 30%) and the IBS-SSS score (> 50 points). The effectiveness of *B. longum* 35624^®^ was rated as “good to satisfactory” by study participants and the investigating physicians. One mild adverse event (nausea) was potentially linked to the study treatment. We conclude that an 8-week course of *B. longum* 35624^®^ was associated with significant, clinically meaningful symptom relief in a typical population of adult patients with IBS.

## Introduction

Irritable bowel syndrome (IBS, also known as functional bowel disorder) is a disorder of the brain-gut-microbiota axis. Although the estimated incidence of this syndrome varies from 4 to 10% (depending on the country and the diagnostic criteria applied), IBS is clearly one of the disorders most frequently diagnosed by gastroenterologists [1, 2]. According to the Rome IV diagnostic criteria, IBS corresponds to recurrent abdominal pain on average at least 1 day per week in the last 3 months, associated with two or more of the following criteria: (i) related to defecation, (ii) a change in frequency of stools, and (iii) a change in form (appearance) of stools [3]. Additional signs and symptoms of IBS typically include bloating, increased passage of gas, diarrhea, and constipation. Most patients suffer from a complex mixture of symptoms that differ in their severity, duration, and rank; hence, the Rome IV diagnostic criteria define four IBS subtypes: diarrhea-predominant (IBS-D), constipation-predominant (IBS-C), mixed (IBS-M), and unclassified (IBS-U) [3]. Although the pathophysiology of IBS has not been fully characterized, recent research suggests that several mechanisms are involved; these include bidirectional dysregulation of brain-gut interactions, interactions between the microbiota, the intestinal barrier and visceral hypersensitivity, psychological disturbances, low-grade intestinal inflammation, permeability, genetic factors, and enteral infections [4].

In view of IBS’s chronic nature, variability, and both intestinal and extraintestinal symptoms, this condition is a major health concern and has a negative impact on quality of life [5]. The main treatment objective is to alleviate the symptoms of IBS and thus improve quality of life. Most of the currently available conventional treatments for IBS are general lifestyle measures (more physical activity, good sleep habits, relaxation, etc.), dietary measures (e.g., the low fermentable oligosaccharide, disaccharide, monosaccharide, and polyol (FODMAP) diet), or medications (such as antispasmodics, laxatives, antidiarrheals, and antidepressants) [6–9]. However, the response rates in randomized controlled trials (RCTs) of medications (~ 40%) are often barely above those achieved with a placebo (20–30%) [10].

People with IBS show alterations in their intestinal microbiota [11]. Imbalances in the microbiota or even dysbiosis might not only produce changes in the gut barrier (thus increasing the latter’s permeability to compounds and microorganisms) but also impair immune responses. It is now acknowledged that greater intestinal permeability is associated with visceral hypersensitivity and thus abdominal pain [5, 12]. Hence, treatments that influence the microbiota and the gut barrier appear to be promising tools for the management of IBS. It has been demonstrated that specific probiotic bacterial strains are able to modulate the microbiota, strengthen the gut barrier, and thereby reduce disruption of the intestinal wall [13]. Furthermore, RCTs have shown that several probiotic strains are clinically efficacious in reducing the frequency and severity of the symptoms of IBS [6, 7, 14–16]. Many (but not all) of today’s guidelines encourage the use of evidence-based probiotics in the treatment of IBS [17–23]. However, given the spectrum of individual symptoms and multifactorial pathophysiology of IBS, a standard treatment (whether probiotic or not) for all affected patients is not available [7].

The natural bacterial strain *Bifidobacterium longum* 35624^®^ was originally isolated from the gut epithelium of a healthy human and is able to survive gastrointestinal transit [13, 24]. *Bifidobacterium longum* 35624^®^ has specific genetic features, one of which leads to the generation of a characteristic exopolysaccharide [25]. Evidence from RCTs has proven the strain’s efficacy (versus placebo) in alleviating the typical symptoms (abdominal pain, bloating, passage of gas, and bowel dysfunction) in patients with IBS [26–28]. Furthermore, a recent observational study of 278 patients with IBS (diagnosed according to the Rome IV criteria) by Sabaté et al. found that 30 days of treatment with *B. longum* 35624^®^ reduced disease severity and improved the patients’ quality of life — particularly among those with the most severe forms of IBS [28]. Several clinical guidelines now mention *B. longum* 35624^®^ as a probiotic strain for which significant positive effects on IBS have been found [4, 22, 29]. The mechanisms through which *B. longum* in general and *B. longum* 35624^®^ in particular reduce the severity and symptoms in IBS have been extensively investigated in both animal models and humans. Firstly, *B. longum* 35624^®^ binds directly to inflamed colonic mucosa and counters the impairment of gut barrier function seen in IBS [30]. Secondly, *B. longum* 35624^®^ modulates the brain-gut axis through the microbiome (composition and metabolism) and the latter’s interactions with the host [31, 32]. Thirdly, *B. longum* 35624^®^ intake is associated with elevated regulatory T cell induction and thus changes in inflammatory mediator production both in and beyond the gut in humans [33, 34].

Preparations of the strain *B. longum*^®^ 35624 are sold as Alflorex^®^ (Biocodex, Gentilly, France) in various European countries. In the European Union, *B. longum*^®^ 35624 was considered until recently to be a medical device but is now treated as a food supplement.

IBS is a highly variable, individualized disease, the signs, and symptoms of which are easily influenced by factors such as a change in diet or physical activity and psychological comorbidities; this must be taken into account in study designs [35]. In RCTs, these factors are strictly controlled. However, the use of non-RCT evidence is rapidly gaining recognition among the scientific community and by health authorities. A panel of representatives of European health authorities recently stated that real-world evidence should be seen as a valuable tool that complements the findings of RCTs [36]. Hence, well-designed studies enable the extension of RCT evidence to the real world, as an important factor for effectiveness, safety, tolerability, and patient satisfaction. The present observational, post-market clinical follow-up study in Germany was designed to gain clinical data on the effectiveness and safety of the routine use of *B. longum*^®^ 35624 in a typical population of patients with IBS. We conducted this post-market clinical follow-up study because (as mentioned above) *B. longum* 35624^®^ was classified as a medical device at the time of study initiation. Given that the European legislation changed during the study’s recruitment period, clinical evidence was no longer required, and so fewer patients were included than initially planned.

## Material and Methods

### Study Design

We performed an open label, observational, post-market clinical follow-up study of the effectiveness, safety, and tolerability of an 8-week course of *B. longum* 35624^®^ (Alflorex^®^ for IBS, Biocodex*)* in a typical population of adults (aged 18 or over) with IBS (as defined by the Rome IV criteria [3]) in Germany. Participants were recruited by 15 family physicians (FPs). There was an initial study visit on day 1. Each week, the participants rated their IBS symptoms (giving a total IBS symptom score (TISS)) and other endpoints via an online questionnaire. A second (follow-up) visit with the investigating FP was scheduled at the end of the course of treatment (day 57). The study was conducted in accordance with the Declaration of Helsinki and the ISO 14155 standard on the Clinical Investigation of Medical Devices for Human Subjects – Good Clinical Practice and with guidance from the Guideline for Good Clinical Practice issued by the International Council for Harmonisation of Technical Requirements for Pharmaceuticals for Human Use. The study was approved by the corresponding local institutional review boards (Baden-Württemberg, Stuttgart; reference F-2021–027, dated April 8^th^, 2021; North Rhine-Westphalia, Düsseldorf; reference 2,021,079, dated March 24^th^, 2021; Saxony, Dresden; reference EK-BR-58/21–1, dated May 18^th^, 2021). All the participants gave their written, informed consent.

### Study Participants

The inclusion criteria were as follows: age 18 or over, a diagnosis of IBS according to the Rome IV criteria [3], ongoing typical symptoms of IBS (i.e., bloating, passage of gas, diarrhea, abdominal pain, and/or constipation), and an FP recommendation of *B. longum* 35624^®^ (prior to and independently of the present study). Since this was an open-label observational post-market clinical follow-up study, no exclusion criteria were set. Study participants were told to maintain their usual diet, physical exercise patterns, and smoking habits.

The original patient recruitment target was calculated as follows. Firstly, a 50-point reduction in the IBS severity scoring system (IBS-SSS) score is considered to be clinically meaningful [35, 37]. With regard to the literature data, we expected that at least 42% of the participants would experience this reduction. Using a two-tailed binomial test, a power of 90%, and an alpha risk of 0.05, we calculated that 171 participants should be recruited. Assuming a 15% drop-out rate, the recruitment target was therefore *n* = 200.

### The Study Product

The study product (Alflorex^®^ for IBS, called “Alflorex^®^ bei Reizdarm” in Germany) is a probiotic preparation containing one billion lyophilized *B. longum* 35624^®^ bacteria per 0.25 g capsule, which also contains excipients (corn starch, hydroxypropyl methylcellulose, and magnesium stearate). At the time of the study, this product was classified as a class IIb medical device under the European Union’s Directive 93/42/EEC and Directive 2007/47/EC. The product is intended to reduce symptoms of IBS, including symptoms of bloating, gas, abdominal pain, diarrhea, and constipation. In accordance with the European Union’s Medical Device Directive 93/42/EEC, this post-market clinical follow-up study was designed to proactively collect clinical data on the efficacy and safety of the daily use of Alflorex^®^ bei Reizdarm by IBS patients under real-life conditions.

### Assessments and Endpoints

The study’s primary endpoint was the change in an IBS total symptom score (TISS). Once a week during the course of treatment with *B. longum* 35624^®^, the study participants rated each of five IBS symptoms (abdominal pain, bloating, gas, difficulty defecating/constipation, and urgency/diarrhea) via an online questionnaire on a 6-point-Likert-scale (0 = no symptoms; 5 = very intense symptoms). The individual symptom scores were then summed to give the TISS. Miller has suggested that a reduction in symptoms of at least 30% (relative to baseline) is clinically meaningful [35].

The study’s secondary endpoints were changes over time in each individual IBS symptom, the severity of the IBS according to the IBS-SSS part I, in which pain, distension, bowel dysfunction, and quality of life/global wellbeing were scored online on days 1, 29, and 57 [37]; interference of the symptoms of IBS with life in general; safety (adverse events during the treatment); overall tolerability (assessed online at the end of the study by the physicians and participants on a 6-point Likert scale ranging from 1 (very good) to 6 (very bad)); and overall effectiveness (also assessed online at the end of the study by the physicians and the participants on a 6-point Likert scale ranging from 1 (very effective) to 6 (ineffective)). Data on interference with life in general were extracted from question 4 of part 1 of the IBS-SSS questionnaire.

### Statistical Analysis

Changes in the TISS during the 8-week course of treatment were analyzed using Friedman’s test. Post hoc analyses of individual time points were performed with Dunn’s test for multiple comparisons. Changes over time in IBS-SSS scores, including the score for interference with life in general, were analyzed with Wilcoxon’s signed rank test. Single missing items of data were imputed using the “last observation carried forward” method, if this use was judged to be reasonable.

## Results

### The Study Population

In all, 37 study participants were enrolled and received at least one dose of the study product during the period from April 2021 to September 2021, forming the intention-to-treat (ITT) population. Four participants had major protocol deviations (failure to complete the weekly online survey, and/or failure to attend the second FP visit) and so the per-protocol (PP) population comprised 33 participants (Table 1). All IBS subtypes were represented in the PP population (Table 1), although the proportion of the IBS-C subtype was notably lower than the value reported in another recent study of *B. longum* 35624^®^ in IBS [28].

**Table 1:** Baseline characteristics of the study participants in the PP population

	The PP population (*n* = 33)
Age (years), mean (range)	41 (19–75)
Men/women^a^, *n* (%)	12/20 (36%/61%)
Time since IBS onset, at the time of inclusion (years)	3.65
IBS-D subtype	10 (30.3%)
IBS-C subtype	5 (15.2%)
IBS-M subtype	9 (27.3%)
IBS-U subtype	7 (21.2%)
IBS-SSS score^b^ and the corresponding grade	236.13 (“moderate”)
Abdominal pain severity^c^	2.36
Bloating severity^c^	3.16
Diarrhea severity^c^	2.42
Constipation severity^c^	1.36
Passage of gas severity^c^	3.03
TISS^d^	12.32

*PP* per-protocol, *IBS* irritable bowel syndrome, *IBS-D* diarrhea-predominant, *IBS-C* constipation-predominant, *IBS-M* mixed, *IBS-U* unclassified, *IBS-SSS* irritable bowel syndrome severity scoring system, *TISS* IBS total symptom score

^a^Missing data: *n* = 1

^b^Possible range: 0–500 (175–300: moderate)

^c^Mean value rated on a 6-point-Likert scale (0 = no symptoms; 5 = very intense symptoms)

^d^The mean sum of the individual symptoms (abdominal pain, bloating, gas, difficulty defecating/constipation, and urgency/diarrhea); range: 0 (no symptoms) to 25 (all symptoms, all very intense)

Twenty-nine of the 33 participants stated that they did not follow a particular diet, one participant was a vegetarian, and one was a vegan (missing data: *n* = 2).

According to the IBS-SSS, the mean severity of the disease at baseline was moderate (mean score: 236.1). Sixteen (48.5%) of the 33 study participants suffered from moderate IBS (again according to the IBS-SSS), 8 (24.2%) suffered from severe IBS, 5 (15.2%) suffered from mild IBS, and 2 (6.1%) were in remission at baseline.

Fourteen (42%) of the participants in the PP population suffered from concomitant diseases: chronic back pain (*n* = 1), palpitations (*n* = 1), hypertension (*n* = 3), exocrine pancreatic insufficiency (*n* = 1), diabetes mellitus type 2 (*n* = 1), anxiety disorder (*n* = 1), colon carcinoma (*n* = 1), polymyalgia rheumatica (*n* = 1), reflux diseases (*n* = 2), hypothyroidism (*n* = 3), asthma (*n* = 1), varicose veins in lower extremities (*n* = 1), supraventricular tachycardia (*n* = 1), urinary tract infection (*n* = 1), multiple sclerosis (*n* = 1), and endometriosis (*n* = 1). The investigating FPs considered that none of these concomitant diseases were likely to directly influence the action of *B. longum* 35624^®^.

Fifteen (45%) of the participants in the PP population were taking one or more concomitant medications. The only medication considered by the investigating FPs to have a potential influence on the action of *B. longum* 35624^®^ was an antibiotic in one patient at baseline, although this was discontinued during the first week of treatment.

### The Primary Effectiveness Criterion: the Total IBS Symptom Score

**Fig. 1:**
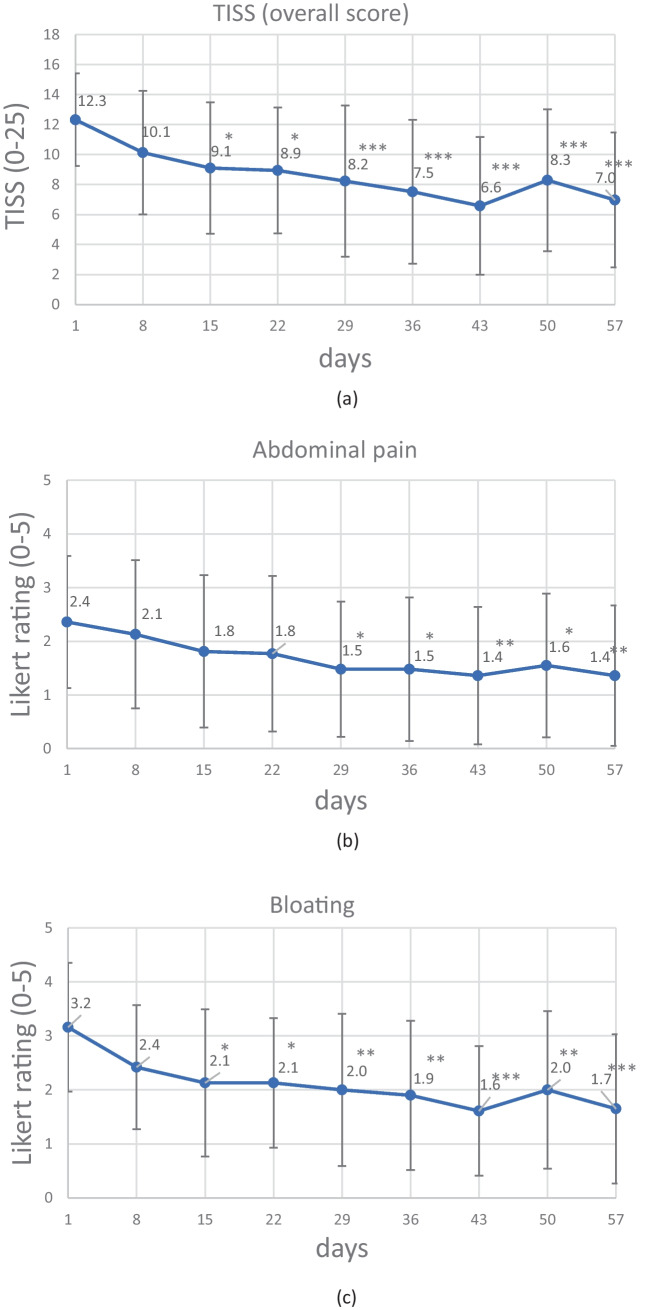

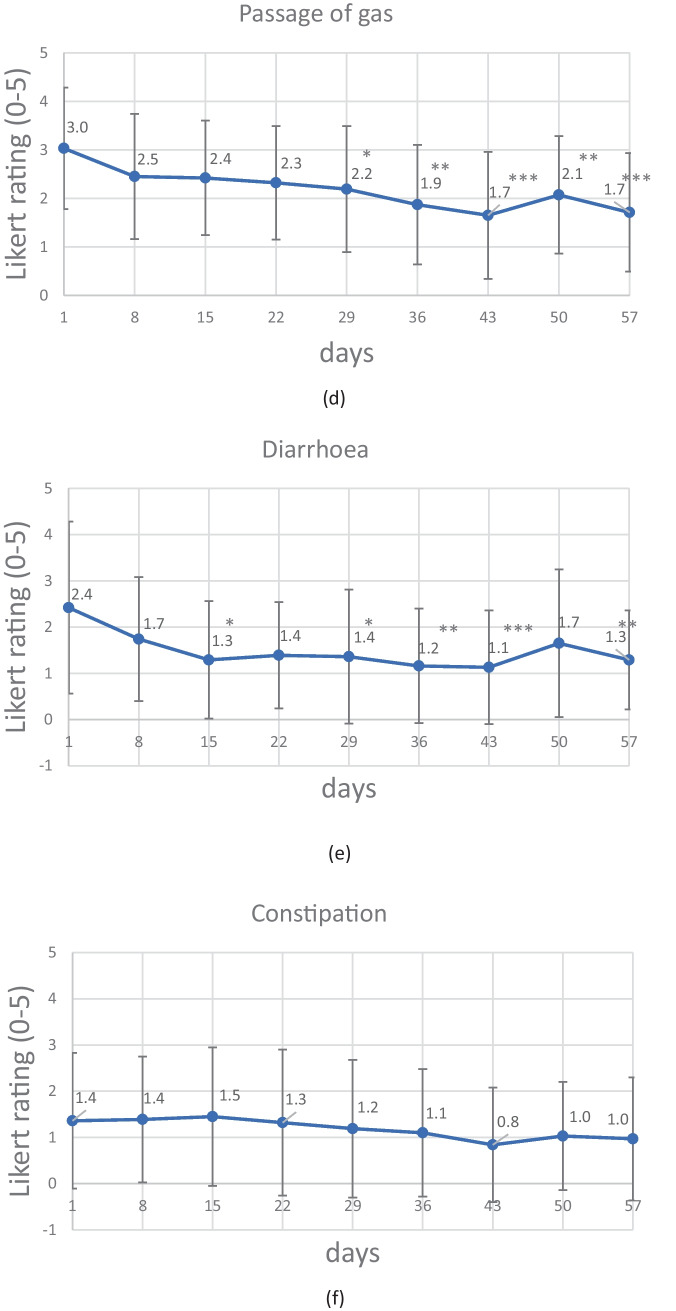
Changes in the TISS (**a**) and the individual IBS symptom scores (**b** to **f**) during the 8-week course of treatment with B. longum 35624^®^ (mean ± SD). Each time point was compared with baseline (day 1) using Dunn’s post hoc test for multiple comparisons; asterisks indicate statistically significant differences (*p < 0.05, **p < 0.01, ***p < 0.001)

As shown in Fig. 1b–f, the individual symptom scores comprising the TISS fell steadily over the 8-week course of *B. longum* 35624^®^. Significant changes vs. baseline were observed for all individual symptoms except constipation, usually after 2 to 3 weeks of treatment. Accordingly, the TISS fell significantly from a mean value of 12.3 at baseline to 8.2 at week 4 and 6.97 at week 8 (a 43.5% reduction; *p* < 0.0001; Fig. 1). A post hoc analysis showed that a significant reduction in the TISS had already been achieved after two weeks (*p* < 0.05; Fig. 1). The lowest mean TISS was observed at week 6.

### The Secondary Effectiveness Criteria

#### The Individual Symptom Scores

The 8-week course of *B. longum* 35624^®^ was associated with statistically significant changes over time in the bloating, passage of gas, diarrhea (*p* < 0.0001 for all three), and abdominal pain (*p* < 0.001) (Fig. 1). The mean score for constipation fell slightly (albeit not significantly) during the course of treatment. A post hoc analysis showed that the scores for bloating and diarrhea had already fallen significantly after 2 weeks of treatment, whereas abdominal pain and passage of gas had fallen significantly after 4 weeks of treatment.

#### A Clinically Meaningful Reduction in the TISS

**Fig. 2:**
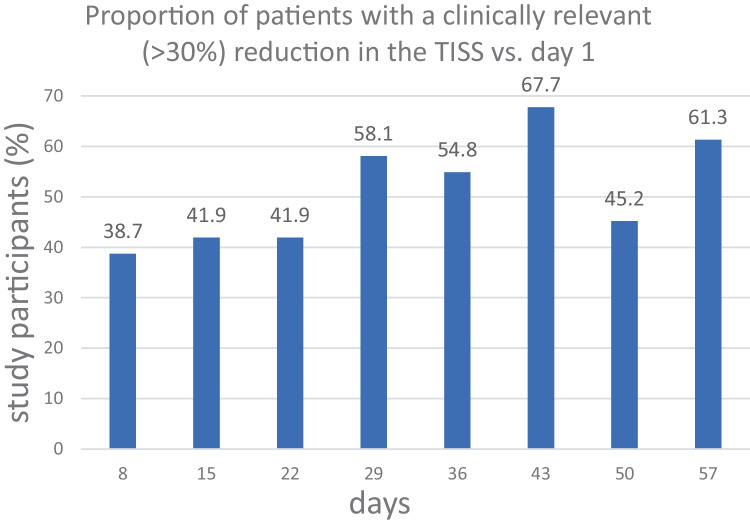
The change over time in the percentage of study participants (PP population) with a clinically relevant (> 30%) reduction vs. baseline in the TISS

After 1 week, a clinically meaningful (> 30%) reduction in the TISS was observed for 38.71% of the study participants (Fig. 2). This proportion peaked at 67.7% after 43 days. By the end of the course of treatment, this percentage was still over 60%.

#### The IBS-SSS Score

**Fig. 3:**
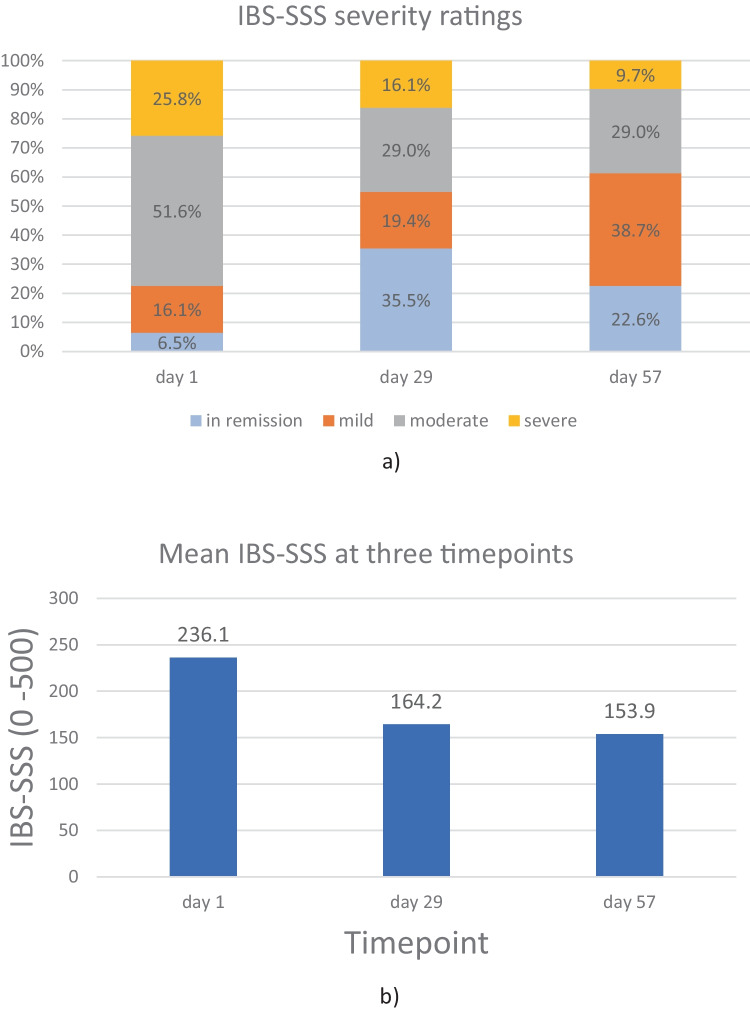
**a** IBS severity ratings (according to the IBS-SSS) at three timepoints, in the PP population (n = 31). IBS-SSS score of 0–75: in remission; 75–174: mild IBS; 175–299: moderate IBS; > 300: severe IBS. **b** The mean IBS-SSS scores at the three timepoints

As shown in Fig. 3a, 77.4% of the study participants suffered from moderate or severe IBS (i.e. an IBS-SSS score > 175) at baseline. This percentage fell to 45.1% on day 29 of the course of *B. longum* 35624^®^ and 38.7% on day 57. The proportion of study participants in remission or with mild IBS (IBS-SSS score < 175) rose from 22.6% at baseline to 54.9% on day 29 and 61.3% on day 57 (Fig. 3a). At 8 weeks, 64.5% of the study participants had achieved a clinically meaningful reduction (> 50 points) in the IBS-SSS score (Fig. 3b). The fall of 82 points (vs. baseline) in the IBS-SSS score over 8 weeks was statistically and clinically significant (236.1 ± 101.7 at baseline *vs*. 153.9 ± 110.0; *p* < 0.001; Fig. 3b).

##### Quality of Life

**Fig. 4:**
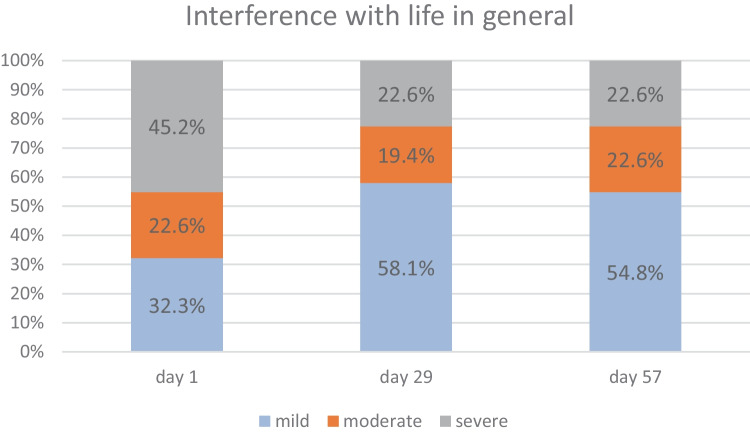
Distribution of the grades for IBS’s interference with life in general (question 4 from part 1 of the IBS-SSS questionnaire) at three timepoints, in the PP population. NB: rounding to a single decimal place means that the total percentage exceeds 100% for days 1 and 29

As shown in Fig. 4, the 8-week treatment with *B. longum* 35624^®^ was associated with a significant reduction in the interference of IBS symptoms with life in general (mean scores: 5.39 on day 1, 3.55 on day 29 (*p* = 0.0028 vs. day 1), and 3.94 on day 57 (*p* = 0.0032 vs. day 1)). The corresponding mean scores were 6, 3, and 3. The percentage of study participants reporting “mild interference of IBS symptoms with life in general” (score between 0 and 30) increased from 32.3% on day 1 to 58.1% on day 29 and 54.8% on day 57 (Fig. 4). Correspondingly, the percentage of study participants reporting “high interference of IBS symptoms with life in general” (a score between 70 and 100) decreased from 45.2% on day 1 to 22.6% on days 29 and 57 (Fig. 4).

#### Overall Effectiveness

Over 60% of the study participants stated that they felt “adequate relief” (relative to the start of the study) after both 4 and 8 weeks of treatment. On day 57, 50% of the study participants rated study product’s effectiveness (on a 6-point-Likert scale) as “very good” or “good,” and 30% rated it as “satisfactory.” On day 57, the investigating physicians rated their perception of the study product’s effectiveness (on the same 6-point-Likert scale) as “very good” or “good” for 64.3% of the participants. For both the study participants and the investigating physicians, the main reasons for effectiveness were “a prolongation of symptom-free periods” and “a reduction in IBS symptoms.”

### Safety and Tolerability

None of the study participants reported any adverse events, either spontaneously during the study or in response to a specific question from the FP at the second study visit. However, written comments on effectiveness and satisfaction made by eight study participants in the day 57 questionnaire were suggestive of the occurrence of an adverse event and were evaluated further. Six of these comments corresponded to a perceived lack of effectiveness and were thus discarded. One study participant reported having more severe constipation during the course of treatment. However, this could have corresponded to a typical fluctuation in IBS symptoms and was not considered to be causally linked to the study product. Lastly, another study participant reported mild nausea, mild abdominal pain, and more severe passage of gas during week 4; we considered that this was the only adverse event with a potential link to the study product.

**Fig. 5:**
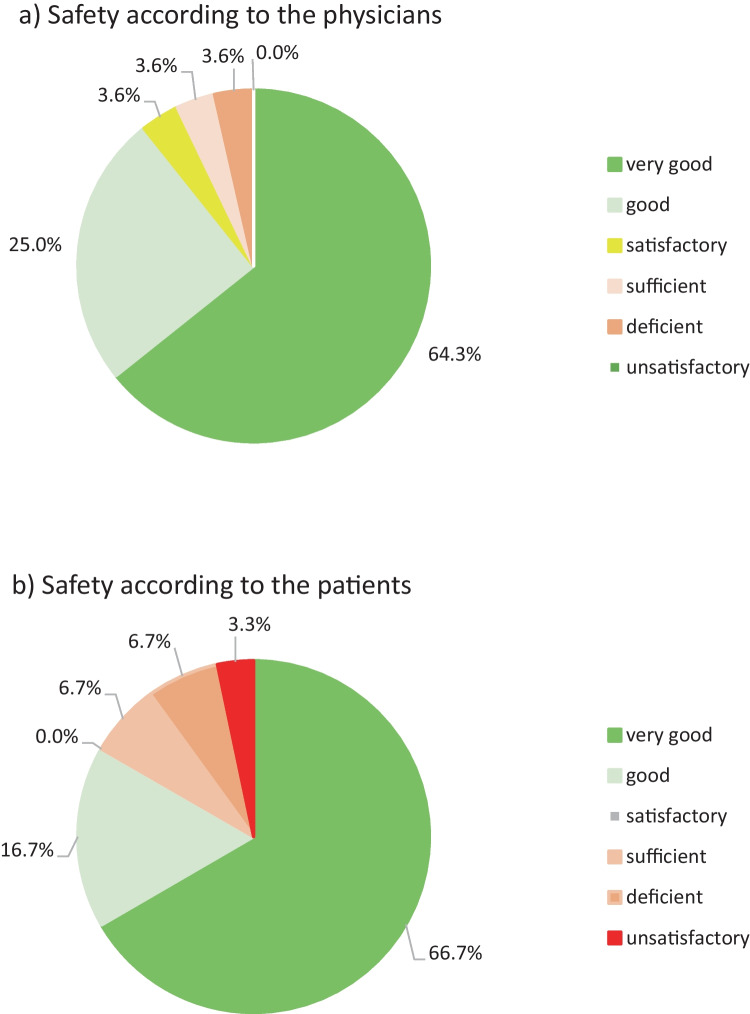
Tolerability of the study product, as rated on day 57 by the investigating physicians and the study participants

Tolerability was evaluated in the ITT population (*n* = 37) (Fig. 5). The study product’s tolerability was rated as “very good” or “good” by the great majority of the study participants (89.3%) and the investigating physicians (83.4%) (Fig. 5). The mean safety score (out of 6, ranging from 1 high to 6 low) was 1.57 according to the investigating physicians and 1.80 according to the study participants.

## Discussion

We performed an open-label, observational postmarketing study of a typical population of patients with IBS in Germany. Although fewer patients were included than initially planned (due to a change in product status), the study was completed and the results provided novel evidence of *B. longum*^®^ 35624’s effectiveness, safety, and tolerability in patients with primarily moderate or severe IBS at baseline.

 According to Miller et al. the use of appropriate clinical study endpoints (i.e. endpoints sensitive to changes in all subtypes of IBS) is of great importance [35]. Subtype-specific symptoms alone (e.g., diarrhea, constipation, or stool consistency) are not suitable for the detection of symptom alleviation in a typical IBS patient population [35]. In the present study, the main effectiveness criterion was a TISS (i.e., the sum of five typical IBS symptoms: abdominal pain, bloating, passage of gas, constipation, and diarrhea). The use of a TISS mitigated the influence of individual, predominant symptoms in certain subtypes. The TISS fell by 43.5% during the 8-week course of treatment with *B. longum* 35624^®^, evidencing a clear effect of the bacterial strain on IBS symptoms.

As the one of the mandatory symptoms for defining IBS (according to the ROME IV criteria), abdominal pain has also been referred to as a suitable study endpoint. Furthermore, the change in the IBS-SSS score and a statement of “adequate relief” (vs. before treatment) are reportedly adequate endpoints in an average IBS population [35]. All three variables were measured in the present study and all showed a significant improvement during the use of *B. longum* 35624^®^*.* Statistical testing of the data on the “adequate relief” variable was not appropriate but more than 60% of the study participants reported having experienced adequate relief after 4 weeks and 8 weeks of treatment.

However, statistically significant improvements are not necessarily clinically meaningful. In patients with IBS, a symptom reduction of more than 30% and a reduction in the IBS-SSS score of more than 50 points (vs. baseline) are generally accepted as being clinically meaningful [35, 37]. We found that 58% of study participants achieved a clinically meaningful improvement (> 30% reduction) in the TISS after only 4 weeks of treatment with *B. longum* 35624^®^ (week 8: 61%). Furthermore, clinically meaningful improvements in abdominal pain and the IBS-SSS score were achieved within 4 weeks of treatment. The mean score for abdominal pain was reduced by ~ 37% and the mean IBS-SSS score was reduced by more than 70 points on day 29 compared to baseline (week 8: − 42% abdominal pain; − 82.2 IBS-SSS points). More than 50% of the study participants achieved a clinically meaningful improvement of IBS-SSS by week 4, and the benefit was present at week 8. We conclude that in this observational setting, *B. longum* 35624^®^ led to a significant and clinically meaningful improvement in the symptoms of IBS within 4 weeks of treatment.

Overall, our results are in line with those of another recent observational study of the same probiotic in a larger number of patients [28]. Sabaté et al. studied 278 patients diagnosed according to the Rome IV criteria and enrolled by private practice gastroenterologists in France. Their study population was not dissimilar to ours with regard to age, the IBS subtype proportions, and the severity of IBS (as also judged by the IBS-SSS). Although Sabaté et al.’s patients received 10^9^ CFU *B. longum* 35624^®^ for 30 days only, the results were very similar to ours at 4 weeks. The researchers observed a clinically meaningful reduction in the IBS-SSS in 65.7% of the patients (vs. 58.1% in the present study) and thus corresponding shifts to lower IBS-SSS grades. There was also a marked improvement in the overall quality of life, as measured with a detailed (34-item) IBS quality of life questionnaire (IBS-QOL). The mean ± SD IBS-QOL score was 60.2 ± 20.5 at baseline and 68.8 ± 20.9 after 4 weeks (*p* < 0.001) [28].

On average, the individual symptom score for bloating, passage of gas, and diarrhea changed significantly from “mild or moderate” at baseline to “very mild or mild” within 2 to 4 weeks of treatment. *B. longum* 35624^®^ appears to be associated with a reduction in all the typical symptoms of IBS, regardless of the predominant individual symptom. In contrast, a clinically meaningful symptom reduction was not observed for constipation; however, this might be because the mean value at baseline was already low (i.e., a floor effect). In addition to the severity of specific IBS symptoms, the impact on general life is a very constraining aspect for patients. On day 1, almost half of the study participants (45.2%) stated that IBS interfered greatly with their general life. This proportion fell to 22.6% on day 29 and also 22.6% on day 57 — evidencing a great improvement in the patients’ quality of life and general wellbeing.

It is well known that IBS trials are subject to a strong placebo effect; the mere fact of being treated can lead to clinical effects [10]. In a somewhat dated meta-analysis of IBS trials, Patel et al. found that “the placebo response ranged from 16.0 to 71.4% with a population-weighted average of 40.2%” [38]. Similarly, Ford and Moayyedi reported that the pooled placebo response rate across all RCTs was 37.5% [39]. Most recently, a systematic review and meta-analysis by Bosman et al. found that on average, 27.3% of patients with IBS experience a placebo response when “global improvement” is used as an endpoint [10]. In our observational study, a “global improvement” corresponded to “adequate relief” (response rate: > 60%) and/or a clinically meaningful reduction in the TISS (response rate: > 60%) [10]. Hence, the response rates in the present study clearly exceeded the typical value for a placebo. One should perhaps not focus unduly on absolute values; indeed, van der Geest et al. commented in their meta-analysis that determining the overall efficacy of probiotic and drug-based interventions in IBS is inherently complex [40]. We nevertheless conclude that 4 weeks of *B. longum* 35624^®^ intakes had a real effect on IBS symptoms in responders and that the treatment effect is still strongly present at 8 weeks.

We found that the incidence of adverse events with a potential causal link to the intake of *B. longum* 35624^®^ was very low (mild nausea, in 1 out of 33 patients). This low incidence is in line with the literature data [26–28]. The clinical trial evidence shows that the intake of *B. longum* 35624^®^ can lead to temporary changes in bowel habits during the first weeks of intake; these changes are not uncommon as the gut adapts to the presence of the bacterial strain [26, 27]. Hence, we considered that the treatment with *B. longum* 35624^®^ had an excellent safety profile. Lastly, the study product’s tolerability was rated as “very good to good” by the investigating physicians and study participants.

The efficacy (*vs*. placebo) of *B. longum* 35624^®^ in alleviating the symptoms of IBS has already been proven in RCTs [26, 27]. Open, observational studies (like the present study) are able to complement the evidence from RCTs [28]. In the randomized, placebo-controlled trial conducted by Whorwell et al., a 4-week course of 1 × 10^8^ CFU *B. longum* 35624^®^ daily was associated with a 38% reduction in abdominal pain (relative to baseline), which is very similar to that found in the present post-market clinical follow-up study (37% on week 4) [26]. In the RCT, the patients in the placebo group achieved a 25% reduction, which corresponds well to the typical placebo effect reported by Bosman et al. [10]. For bloating and passage of gas, very similar reductions were achieved after 4 weeks of *B. longum* 35624^®^ intakes in the RCT and in our study (bloating: 29% *vs*. 37%, respectively; passage of gas: 23% *vs*. 28%, respectively). However, our present results in a typical IBS population showed that the symptom reduction was even greater after 8 weeks of treatment (a 48% reduction in bloating and a 44% reduction in passage of gas) than after 4 weeks.

The study had a number of strengths. Firstly, we included patients diagnosed according to the Rome IV criteria; with the exception of the work by Sabaté et al. [28], previous studies of *B. longum* 35624^®^ applied the Rome III criteria [26, 27]. In the present observational study, we did not set any exclusion criteria or restrictions regarding diet, lifestyle, or comorbidities. The study participants suffered from various comorbidities, including psychological disorders, hypothyroidism, hypertension, and other diseases that are common in patient populations. Hence, we consider that our study population was representative of adults with IBS of all ages, habits, and subtypes (with the possible exception of IBS-C, which was slightly underrepresented here) and thus matched the conditions for the transfer of evidence on *B. longum* 35624^®^ from RCTs into the real world. Secondly, we used a validated instrument (the IBS-SSS) to measure the severity of IBS. Thirdly, our use of a TISS mitigated the influence of individual, predominant symptoms in certain IBS subtypes. Fourthly, we simultaneously assessed a variety of metrics: overall severity, overall symptoms, individual symptoms, quality of life, safety, tolerability, and overall satisfaction.

The study also had some limitations. Firstly, the study did not include a placebo group or another comparator group, although this is not unusual for an observational study. Secondly, the study’s efficacy endpoints were patient-rated subjective disease scores (the TISS and the IBS-SSS), rather than objective biochemical markers. However, IBS is above all a functional disease, the follow-up of which in primary care is based on the patient’s feelings and symptoms. Furthermore, the IBS-SSS is widely used in clinical studies of IBS [35, 37, 41, 42], and the German-language version was validated by Betz et al. in 2013 [43]. Thirdly, the number of study participants was smaller than planned; this was due to a change in the regulatory situation in Europe during the recruitment period. Nevertheless, the study provided valuable data on the effectiveness, safety, and tolerability of treatment with *B. longum* 35624^®^. Fourthly, the study did not include a post-treatment follow-up period; it would have been interesting to monitor the various endpoints for several weeks after discontinuation of the treatment with *B. longum* 35624^®^. Fifthly, the majority of the study questionnaires were developed in-house and had not been externally validated. For example, we did not administer the IBS-QOL, and so did not collect detailed information on quality of life. However, as mentioned above, we administered the validated IBS-SSS as an index of severity. Sixthly, the study was performed in Germany, and the participants’ ethnicity was not assessed; hence, the present results cannot necessarily be extrapolated to other countries/populations.

## Conclusions

The results of this observational post-market clinical follow-up study suggest that an 8-week course of *B. longum* 35624^®^ is effective and well tolerated in a real-world population of adult patients with IBS (i.e., individuals with different lifestyles, comorbidities, and comedications). Intake of *B. longum* 35624^®^ was associated with high, clinically meaningful response rates (> 60% at 8 weeks, i.e., markedly greater than the mean placebo-group response rate in IBS RCTs). Additional observational studies with larger numbers of patients might further substantiate the level of real-world evidence for *B. longum* 35624^®^ in IBS. In terms of research perspectives, it would be interesting to sample and analyze the participants’ microbiota to see whether the subjective reductions in symptom intensity are associated with objective biological changes in the gut.

## Data Availability

The data that support the findings of this study are not openly available (due to patient confidentiality and consent for limited re-use) but reasonable requests may be sent to the corresponding author.
